# Serial D-dimer measurements dynamically predict disease severity in acute biliary pancreatitis: a prospective observational study

**DOI:** 10.55730/1300-0144.6106

**Published:** 2025-10-30

**Authors:** Kemal TEKEŞİN, Tolga CANBAK, Aylin ACAR, Fatih BAŞAK, Hasan KUMRU, Olgun ERDEM

**Affiliations:** Department of General Surgery, University of Health Sciences, Ümraniye Training and Research Hospital, İstanbul, Turkiye

**Keywords:** Acute pancreatitis, D-dimer, disease severity, Ranson criteria, BISAP score, biomarker

## Abstract

**Background/aim:**

Early risk stratification is required in cases of acute biliary pancreatitis (ABP). Traditional scoring systems such as Ranson’s criteria are complex and often delay treatment. D-dimer, a basic marker of systemic inflammation and coagulation, has shown promise as a prognostic tool. The present study investigates whether the predictive accuracy of serial serum D-dimer measurements is superior to that of the Ranson and BISAP scoring systems for moderate-to-severe ABP.

**Materials and methods:**

Included in this single-center prospective observational study were 264 patients diagnosed with ABP between July 2022 and July 2025 whose collected data were analyzed. The participants were categorized as mild, moderate, or severe based on the current Revised Atlanta Classification (2012), and D-dimer levels were measured at admission (H0), 24 h (H24), and 48 h (H48), along with their BISAP scores to allow a comparative analysis.

**Results:**

Mean D-dimer levels increased significantly with disease severity at all timepoints (p < 0.001). ROC analysis identified the D-dimer level at H48 to have the highest discriminative value for predicting moderate-to-severe ABP (AUC: 0.812; 95% CI: 0.758–0.866). The H24 and H0 D-dimer levels also performed well (AUCs: 0.728 and 0.719, respectively). In comparison, the Ranson (H48 AUC: 0.741) and BISAP scores (H24 AUC: 0.755) yielded lower predictive accuracy – the H48 D-dimer AUC being statistically superior to both (p < 0.05).

**Conclusion:**

Serum D-dimer levels are significantly associated with ABP severity and show promise as a practical, accessible, and cost-effective adjunctive biomarker for early risk assessment. Serial measurements, especially at 48 h, offer superior diagnostic accuracy compared to Ranson and BISAP scoring alone. The clinical measurement of D-dimer levels at 48 h can thus be considered an accessible and timely triage approach. Multicenter prospective validation studies are needed to confirm the diagnostic thresholds and to assess clinical integration.

## 1. Introduction

Acute pancreatitis (AP) is an inflammatory pancreatic condition, the severity of which can range from mild, self-resolving episodes to more severe forms with the potential to lead to organ failure, pancreatic necrosis, and mortality. According to recent multicenter studies, the incidence of acute pancreatitis is continuing to rise globally, with varying trends reported in Europe, Asia, and North America [1].

The most common causes of AP include gallstones and alcohol use [2]. In Türkiye, gallstones are the leading cause of AP [3]. Severe acute pancreatitis (SAP) affects approximately 20% of patients and is associated with a high mortality rate, exceeding 30% in some studies [4]. The early identification of patients at risk for SAP is crucial for the guidance of timely interventions that may improve patient outcomes [5].

The clinical severity of AP is often assessed using such scoring systems as Ranson’s criteria, APACHE II, and BISAP, and morphological indices like the Balthazar Computed Tomography Severity Index (CTSI). While widely used, these systems can be complex, requiring the measurement of multiple clinical and laboratory parameters, potentially leading to delays in decision-making and treatment [5]. Previous comparative analyses have highlighted the limitations of classical scoring systems such as Ranson’s criteria and APACHE II, and have proposed newer indices like BISAP or CTSI to improve early risk stratification [6,7]. In resource-limited centers, where access to advanced imaging or monitoring tools is restricted, this poses an even greater challenge.

The search for simple, cost-effective, and easily accessible biomarkers is thus continuing, and various biomarkers, such as C-reactive protein (CRP), neutrophil-to-lymphocyte ratio (NLR), platelet-to-lymphocyte ratio (PLR), antithrombin III, and D-dimer, have been investigated as potential predictors of disease severity. It has been demonstrated that combining CRP with Ranson’s criteria enhances its efficacy in predicting SAP [8], while antithrombin III and D-dimer have been tested in hyperlipidemic and biliary pancreatitis patients, demonstrating potential relevance across various subgroups [9]. D-dimer, in particular, offers a significant advantage, as elevated levels point to underlying fibrin degradation, secondary to widespread endothelial activation, intravascular coagulation, and systemic inflammation, all of which are central to the pathophysiology of severe AP [10]. This approach not only captures both coagulation abnormalities and systemic inflammation, but is also widely accessible, inexpensive, and practical for use when advanced imaging modalities or intensive care infrastructures are lacking. Furthermore, D-dimer’s proven value as a prognostic marker in other inflammatory and thrombotic conditions, such as sepsis and severe trauma, reinforces its promise for AP [10].

The present study investigates the diagnostic utility of serial serum D-dimer measurements as a simple, dynamic biomarker for early severity prediction in ABP, and compares its performance to both the Ranson scoring system and the more contemporary BISAP score.

## 2. Materials and methods

Ethical approval was obtained from the University of Health Sciences, Ümraniye Training and Research Hospital Clinical Research Ethics Committee (approval number: 157, date: April 21, 2022). The study was conducted in accordance with the Declaration of Helsinki, and all participants provided written informed consent for the use of their data prior to publication.

**Figure 1:**
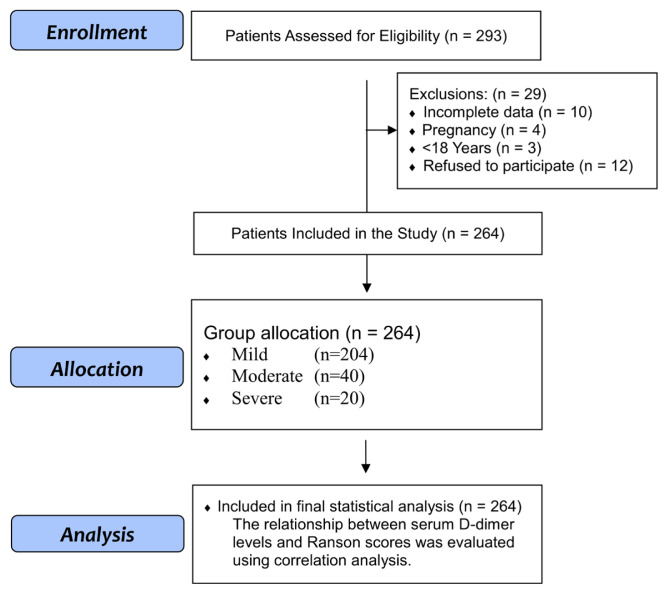
Flow diagram of patient selection and study inclusion criteria.

In this single-center, prospective observational study, data were collected prospectively from patient admissions and subjected to subsequent analysis. Included in the study were 264 patients diagnosed with ABP and hospitalized in the Department of General Surgery, University of Health Sciences, Ümraniye Training and Research Hospital between July 1, 2022, and July 1, 2025. No formal sample size calculation was performed; however, a post hoc power analysis indicated that the sample of 264 patients was sufficient for the detection of moderate correlations (r > 0.3) with 80% power at α = 0.05. Figure 1 presents a flow diagram of the study detailing the patient selection and exclusion criteria, and the number of participants assessed at each stage of the study.

Included in the study were patients aged ≥ 18 years who had been diagnosed with ABP, while those < 18 years of age, pregnant, those with known underlying coagulopathies (e.g., DIC), or pre-existing thromboembolic disease (e.g., deep vein thrombosis or pulmonary embolism), those receiving oral or injectable anticoagulant therapy at the time of admission, or those who were unwilling to participate in the study were excluded. Patients with concomitant infectious processes such as pneumonia, which could independently elevate D-dimer, were also excluded from the analysis.

Diagnoses were confirmed based on the Revised Atlanta Classification (2012) [11]. The severity of ABP was categorized according to the Revised Atlanta score as mild (No organ failure, no local/systemic complications), moderate (Transient organ failure resolves within 48 h and/or local/systemic complications without persistent organ failure), or severe (single or multiple organ failure persisting for more than 48 h).

Serum D-dimer levels were recorded at admission (H0), at the 24th h (H24), and at the 48th hour (H48). The demographic data, comorbidities, admission date, and the number and presence of previous or recurrent pancreatitis attacks of each patient were recorded. Also noted were serial D-dimer values, Ranson’s criteria scores at admission and at H48, BISAP scores at H24, early and overall cholecystectomy status, length of hospital stay, intensive care unit (ICU) admission, and mortality status.

### 2.1. Statistical analysis

Normality of continuous variables was assessed using the Shapiro–Wilk test. Continuous variables with a normal distribution are expressed as mean ± standard deviation (SD), while those with non-normal distribution were summarized as median (minimum-maximum). As the majority of variables did not follow a normal distribution, non-parametric statistical tests were employed for analysis. Spearman correlation coefficients were calculated to assess the relationship between D-dimer levels and severity scores. The sensitivity and specificity of D-dimer levels in predicting severity were assessed using Receiver Operating Characteristic (ROC) analysis. Area Under the Curve (AUC) values were presented with 95% Confidence Intervals (CI), for which mild ABP cases were compared with combined moderate and severe ABP cases. The Youden Index was calculated for Ranson and BISAP scores and D-dimer levels at multiple time points to assess their diagnostic performance. A formal comparison of the AUCs of D-dimer and Ranson/BISAP was performed using DeLong’s test.

For comparisons across the three severity groups (mild, moderate, and severe), categorical variables were analyzed using the chi-square test or Fisher’s exact test, as appropriate. Continuous variables were compared using the Kruskal-Wallis test due to their non-normal distribution. Post-hoc pairwise comparisons were conducted where appropriate using Dunn’s test with Bonferroni correction. A p-value of < 0.05 was considered statistically significant. All statistical analysis and data visualizations were performed using IBM SPSS Statistics, Version 26.0. (Armonk, NY: IBM Corp., 2019).

## 3. Results

The baseline characteristics of the 264 patients included in the study are presented in Table 1, stratified by severity group. According to the Revised Atlanta Classification, 204 patients (77.2%) were classified as mild, 40 (15.1%) as moderate, and 20 (7.5%) as severe. Age differed significantly among the severity groups (p < 0.001, Table 1). The mean ages in the moderate and severe cases were similar, while the mean age in the mild group was lower (57 years for mild, 62.5 years for moderate, and 62 years for severe) (Table 1).

Mean serum D-dimer levels showed a progressive increase with disease severity at H0, H24, and H48 h (Table 2). A positive correlation was found between disease severity and D-dimer levels at all timepoints (Table 2).

Kruskal–Wallis analysis demonstrated a significant difference in D-dimer levels among the severity groups at all timepoints (Table 2). In pairwise comparisons, significant differences in D-dimer levels were noted between the mild and moderate groups (Test Statistic = −37.155, p = 0.013), and between the mild and severe groups (Test Statistic = −63.230, p = 0.001) at H0. At H24, the mild group differed significantly from both the moderate (Test Statistic = −42.290, p = 0.003) and severe groups (Test Statistic = −66.415, p < 0.001). Similarly, at H48, significant differences were observed between the mild and moderate (Test Statistic = −63.752, p < 0.001) and mild and severe groups (Test Statistic = −91.740, p < 0.001). In contrast, no significant differences were noted between the moderate and severe groups at any time point (all p > 0.05 after Bonferroni correction). While D-dimer levels significantly differentiated the mild cases from the moderate and severe groups, the lack of statistical separation between the moderate and severe cases suggests a plateau effect in D-dimer elevation.

**Figure 2:**
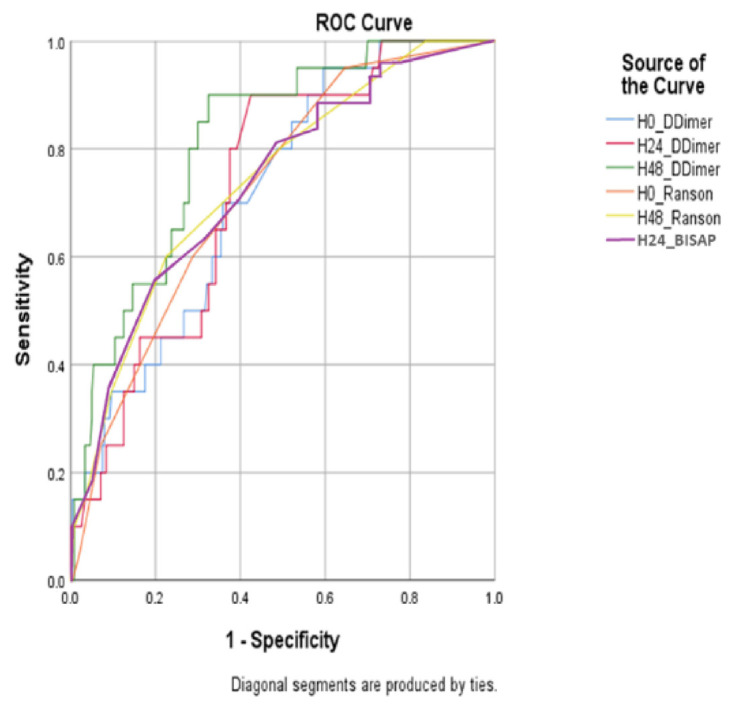
Receiver operating characteristic (ROC) curves of D-dimer levels and Ranson/BISAP scores at different time points. ROC curves demonstrating the diagnostic accuracy of D-dimer concentrations and Ranson/BISAP scores measured at admission (H0), 24 h (H24), and 48 h (H48). Among all parameters, H48 D-dimer exhibited the highest area under the curve (AUC: 0.812, 95% CI: 0.758–0.866), indicating superior discriminative performance. The optimal cut-off value for H48 D-dimer (1961 ng/mL) yielded a sensitivity of 0.95 and specificity of 0.467, corresponding to the highest Youden Index (0.417). In contrast, Ranson scores showed demonstrated lower diagnostic utility, particularly at H48 (Youden Index: 0.375), and the H24 BISAP score (AUC: 0.755) also performed lower than H48 D-dimer. Shaded areas represent 95% Confidence Intervals for each curve. Diagonal segments indicate tied values in the data.

ROC curve analysis revealed the H48 D-dimer level offered the highest overall diagnostic accuracy (AUC: 0.812, 95% CI: 0.758–0.866)), while D-dimer at H24 recorded the highest Youden Index (0.475; sensitivity: 0.900; specificity: 0.575), indicating the best sensitivity–specificity balance. H0 D-dimer showed moderate performance (AUC: 0.719, 95% CI: 0.660–0.778; Youden Index: 0.338). In comparison, Ranson’s criteria demonstrated lower utility, with H0 and H48 AUCs of 0.723 (95% CI: 0.664–0.782) and 0.741 (95% CI: 0.683–0.799), and Youden Indices of 0.313 and 0.375, respectively. The BISAP score at H24 yielded an AUC of 0.755 (95% CI: 0.698–0.812) with a Youden Index of 0.400. DeLong’s test confirmed the AUC for D-dimer at H48 to be significantly higher than that of Ranson’s criteria at the same time point (p = 0.038). These findings are detailed in Table 3 and illustrated in Figure 2.

## 4. Discussion

ABP encompasses a wide spectrum of disease severity, and the timely identification of severe cases is critical to optimizing outcomes [2]. Traditional scoring systems, such as Ranson’s criteria, are helpful for assessing disease severity but rely on the measurement of multiple clinical parameters, which may delay risk stratification [5].

In the present study, patients were divided into three groups according to disease severity as mild, moderate, or severe, using the Revised Atlanta Classification [13]. A progressive increase in serum D-dimer levels was observed with increasing disease severity at H0, H24, and H48. These results concur with those of previous studies, and suggest that elevated D-dimer levels reflect the systemic inflammation and coagulation abnormalities seen in severe AP [9]. Moreover, two recent prospective studies similarly identified D-dimer as a viable marker of disease severity, reporting significantly higher levels in patients with complicated AP, including those with pancreatic necrosis and persistent organ failure [14,15].

Spearman correlation analysis demonstrated a significant positive correlation between D-dimer levels and severity across all timepoints, while Kruskal–Wallis and pairwise comparisons revealed that the differences were driven primarily by significantly higher D-dimer levels in the mild group compared to the moderate and severe groups. These differences between the moderate and severe groups, however, were not statistically significant after Bonferroni correction. While D-dimer levels increase with severity, the ability to distinguish moderate from severe AP using D-dimer alone may be limited. This could indicate that D-dimer reaches a threshold of elevation beyond which further severity differentiation becomes less precise, possibly due to overlapping inflammatory cascades.

ROC analysis revealed that D-dimer at H48 offered the highest overall diagnostic performance (AUC: 0.812), while the highest Youden Index was achieved at H24 (0.475), indicating the best sensitivity-specificity balance. Admission D-dimer and Ranson scores at both time points demonstrated lower AUCs and Youden indices, supporting the superiority of serial D-dimer measurements for predicting moderate-to-severe ABP. DeLong’s test confirmed the superiority of H48 D-dimer’s AUC over Ranson’s criteria at H48 and BISAP score at H24. These findings suggest that serial measurements of D-dimer, particularly at H48, offered greater prognostic utility than Ranson’s criteria alone. This reinforces prior evidence suggesting that coagulation markers evolve dynamically over the disease course and may provide more accurate risk stratification when measured over time [13,16,17]. The added value of tracking D-dimer trends over fixed time points is apparent and may guide clinicians in anticipating severe outcomes. Given the high sensitivity and moderate specificity of D-dimer at H48, this marker may be particularly valuable in ruling out severe cases in low-resource or early triage settings.

### 4.1. Comparison with other biomarkers and scores

Our finding of superior performance for H48 D-dimer (AUC 0.812) when compared to the H48 Ranson (AUC 0.741) and H24 BISAP (AUC 0.755) scores is a key finding, showing Ranson’s criteria to be outdated and BISAP remaining a standard for early risk assessment. Specifically, the dynamic change and peak at H48 likely capture the full extent of the systemic inflammatory response and microcirculatory compromise characteristic of severe AP [15]. While our study design did not include a comparison with C-reactive protein (CRP) – the established optimum predictor at 48 h – or procalcitonin, our findings align with those of other studies reporting D-dimer to be an effective prognosticator [16, 18].

Our results further suggest a link between severe pancreatitis and the activation of coagulation pathways and widespread inflammation, both of which contribute to elevated D-dimer levels. The use of D-dimer monitoring alongside existing scoring systems could support early risk stratification and guide clinical decision-making in acute cases.

This study has several limitations, primarily its single-center design, which may limit the generalization of the results. The number of patients in the severe group was relatively small (n = 20, corresponding to 7.5% of the cohort), which could affect the statistical power of the study. Crucially, the study lacks a head-to-head comparison with other contemporary markers and such scoring systems as CRP, NLR, APACHE II, and CTSI, which are considered more contemporary and clinically integrated for severity assessment [6, 7]. Additionally, potential confounders such as pre-existing coagulopathies or anticoagulant use were not taken into account, which could influence D-dimer levels independently of pancreatitis severity [18]. The impact of these factors on D-dimer measurements in our cohort should not be ignored, and represents a source of potential bias. All the above limitations should be considered when interpreting our results.

In conclusion, our study suggests that serum D-dimer levels are significantly correlated with disease severity in acute biliary pancreatitis, and may serve as a practical, accessible, and cost-effective adjunctive biomarker for early risk assessment. Among the tested timepoints, D-dimer at H48 demonstrated the highest diagnostic accuracy. The serial measurement of D-dimer levels, particularly up to H48, may enhance predictive accuracy when used alongside traditional scoring systems such as Ranson’s criteria and BISAP. The clinical utility of a D-dimer threshold (e.g., 1961 ng/mL at H48) lies in its potential for quick and low-cost identification of patients needing aggressive care. Future prospective, multicenter studies incorporating larger severe AP cohorts, additional biomarkers such as CRP and APACHE II, and multivariate modeling are warranted to validate D-dimer’s suggested clinical utility and to identify actionable threshold values for integration into severity stratification protocols. Finally, multicenter validation is essential before D-dimer thresholds are adopted for routine clinical triage.

## Figures and Tables

**Table 1:** Baseline characteristics of patients stratified by pancreatitis severity.

Variable	Mild (n = 204)	Moderate (n = 40)	Severe (n = 20)	p-value	Test
**Age, years**	57 (24–92)	62.5 (28–81)	62 (55–92)	<0.001	Kruskal–Wallis
Rank Mean	120.9	150.21	186.33		
**Sex, n (%)**				<0.001	Chi-square
Male	162 (79.4%)	21 (52.5%)	13 (65.0%)		
Female	42 (20.5%)	19 (47.5%)	7 (35.0%)		
**Hospital Stay, days**	4 (1–17)	4 (2–8)	7 (3–20)	<0.001	Kruskal–Wallis
Rank Mean	122.6	137.07	196.08		
**Early Cholecystectomy**				0.02	Fisher’s Exact
Yes	119 (58.3%)	16 (40.0%)	7 (35.0%)		
No	85 (41.6%)	24 (60.0%)	13 (65.0%)		
**Mortality, n (%)**	0 (0.0%)	0 (0.0%)	6 (30.0%)	<0.001	Fisher’s Exact

**Table 2:** Relationship between D-dimer levels and pancreatitis severity.

	Correlation		Comparison				
Time Point	Spearman’s rho	p-value	Mean Rank			Kruskal–Wallis H	p-value
			Mild (n = 204)	Moderate (n = 40)	Severe (n = 20)		
**H0 D-Dimer**	0.265	<0.001	119.92	157.08	183.15	18.77	<0.001
**H24 D-Dimer**	0.289	<0.001	118.89	161.18	185.3	22.05	<0.001
**H48 D-Dimer**	0.418	<0.001	113.64	177.39	205.38	45.45	<0.001

**Table 3:** Comparison of diagnostic performance of D-dimer and Ranson scores at different time points.

	Cut-off	Sensitivity	Specificity	Youden Index	Area Under Curve	95% Confidence Interval
**H0 D-dimer**	2453 **(ng/mL)**	0.7	0.638	0.338	0.719	0.660–0.778
**H24 D-dimer**	2237.5 **(ng/mL)**	0.9	0.575	0.475	0.728	0.669–0.787
**H48 D-dimer**	1961 **(ng/mL)**	0.95	0.467	0.417	0.812	0.758–0.866
**H0 Ranson Score**	1.5	0.6	0.713	0.313	0.723	0.664–0.782
**H48 Ranson Score**	2.5	0.6	0.775	0.375	0.741	0.683–0.799
H24 BISAP Score	1.5	0.750	0.650	0.400	0.755	0.698–0.812

Cut-off values derived using the Youden Index to optimize diagnostic balance. AUC, sensitivity, and specificity demonstrate performance in identifying moderate-to-severe ABP. Among all parameters, D-dimer at H48 yielded the highest predictive accuracy. H48 D-dimer AUC was significantly superior to H48 Ranson and H24 BISAP (DeLong’s test, p < 0.05).
